# Interactive effects of juvenile defoliation, light conditions, and interspecific competition on growth and ectomycorrhizal colonization of *Fagus sylvatica* and *Pinus sylvestris* seedlings

**DOI:** 10.1007/s00572-015-0645-4

**Published:** 2015-05-24

**Authors:** Lidia K. Trocha, Ewa Weiser, Piotr Robakowski

**Affiliations:** Institute of Dendrology, Polish Academy of Sciences, Parkowa 5, 62-035 Kórnik, Poland; Department of Forestry, Poznan University of Life Sciences, Wojska Polskiego 71E, 60-625 Poznań, Poland

**Keywords:** Ectomycorrhiza, Light environment, Defoliation, Root loss, Juvenile stage of growth, Sink-source relations, Nitrogen acquisition

## Abstract

Seedlings of forest tree species are exposed to a number of abiotic (organ loss or damage, light shortage) and biotic (interspecific competition) stress factors, which may lead to an inhibition of growth and reproduction and, eventually, to plant death. Growth of the host and its mycorrhizal symbiont is often closely linked, and hence, host damage may negatively affect the symbiont. We designed a pot experiment to study the response of light-demanding *Pinus sylvestris* and shade-tolerant *Fagus sylvatica* seedlings to a set of abiotic and biotic stresses and subsequent effects on ectomycorrhizal (ECM) root tip colonization, seedling biomass, and leaf nitrogen content. The light regime had a more pronounced effect on ECM colonization than did juvenile damage. The interspecific competition resulted in higher ECM root tip abundance for *Pinus*, but this effect was insignificant in *Fagus*. Low light and interspecific competition resulted in lower seedling biomass compared to high light, and the effect of the latter was partially masked by high light. Leaf nitrogen responded differently in *Fagus* and *Pinus* when they grew in interspecific competition. Our results indicated that for both light-demanding (*Pinus*) and shade-tolerant (*Fagus*) species, the light environment was a major factor affecting seedling growth and ECM root tip abundance. The light conditions favorable for the growth of seedlings may to some extent compensate for the harmful effects of juvenile organ loss or damage and interspecific competition.

## Introduction

Seedlings of forest tree species are continuously exposed to a set of biotic (grazing, competition with other plants) and abiotic (water deficit, shading) stressors. A prolonged stress may be destructive for a plant when it overcomes plant tolerance. Repetitive plant damages may lead to unsuccessful reproduction and growth and, eventually, to plant death (Clark 1956; Myers 1981).

The effects of plant damages vary depending on the species, organ, and spatial distribution of damage within an individual plant (Maschinski and Whitham 1989). It remains poorly known whether plants may more easily overcome damages that are more distributed throughout the plant or concentrated in one organ (Avila-Sakar and Stephenson 2006). Additionally, plant age is a crucial factor influencing susceptibility to damages like grazing (McNaughton 1983; Muro et al. 1998). For seedlings growing under the tree canopy, the final level of damage also depends on the light conditions, e.g., animals may avoid seedlings growing in the shade (Lincoln and Mooney 1984).

Growth of the host and its mycorrhizal fungus symbiont is often closely linked, and hence, host damage may negatively affect the symbiont. The essential role of ectomycorrhizal fungal symbionts is reflected particularly in the supply of water (Davies et al. 1996; Shi et al. 2002) and nutrients (Perez-Moreno and Read 2000; Lilleskov et al. 2002; Baxter and Dighton 2005), while the fungus requires photosynthates to maintain its functions (Hampp et al. 1999). Carbon flux from host foliage is crucial to maintain ectomycorrhizal fungi (EMF). From 1 to 21 % of the net photosynthetic production is allocated to EMF (Hobbie 2006), and therefore, any aboveground damage may have an impact on root fate and EMF. The effects of damage of these organs may be important for ectomycorrhizal (ECM) morphotypes (both high carbon and low carbon demanding, Saravesi et al. 2008) and EMF species composition (Saikkonen et al. 1999; Kuikka et al. 2003) and, thus, plant maintenance and function. On the other hand, mycorrhizal species differ in their metabolic activities as shown for certain arbuscular mycorrhizal (AM) fungi (Lerat et al. 2003; Bever et al. 2009) and ECM fungi (Pena et al. 2010; Trocha et al. 2010). Thus, carbohydrate depletion may lead to a shift in composition of the mycorrhizal species toward less mutualistic species (Saravesi et al. 2008; Bever et al. 2009) as well as decline of sporocarp production as an effect of restriction of host resources to preserve ECM root tip colonization at an unchanged level (Kuikka et al. 2003). However, defoliation did not decrease the proportion of living ECM root tips (Saikkonen et al. 1999; Kuikka et al. 2003), suggesting a functional balance between carbon sources in plant foliage and belowground sink, i.e., roots and ectomycorrhizae (Saikkonen et al. 1999). In addition, defoliation may hinder whole root system development (Chapin and Slack 1979) due to transient declines in carbon allocation to fine roots (Kosola et al. 2001). Although several studies have examined the effect of carbon shortage on the fate of ECM root colonization (Saikkonen et al. 1999; Kuikka et al. 2003; Markkola et al. 2004; Saravesi et al. 2008), none have examined defoliation and/or root loss that occurs at a very young stage of seedling development. Young seedlings are more threatened by damage than saplings and older trees. Thus, their defoliation and root loss may cause high mortality and a decrease in tree species diversity.

Numerous studies have shown that a reduction of light leads to a decrease in plant biomass (e.g., Tester et al. 1986; Welander and Ottosson 1998). Bücking and Heyser (2003) demonstrated that in low light, *Pinus sylvestris* seedlings inoculated with the ECM fungus *Suillus bovinus* decreased P acquisition and P transfer to the host plant, confirming the role of carbohydrates in mycorrhizal function (Nehls et al. 2010). Shaded plants have lower root biomass compared to high-light conditions (Lambers et al. 1998). This observation suggests that in low-light conditions, plants allocate proportionally less carbon to the roots. Instead, those plants may invest more in leaf production to compensate for light shortage (Bloom et al. 1985 in: Bryla and Eissenstat 2005). However, this statement may be biased because of unequal size when plants are grown under different light conditions (Bryla and Eissenstat 2005). Also, plants experiencing defoliation or grazing on leaves can respond with an increase of photosynthesis in the remaining leaves (Oleksyn et al. 1998; Little et al. 2003).

Closely neighboring plants compete for soil resources. This rule is valid for both individuals of the same species (Facelli et al. 1999), different plant species, and plants with different nutrient needs (Pedersen et al. 1999; McHugh and Gehring 2006). Availability of mycorrhizal fungal inoculum strongly influences plant biomass under different plant densities. Studies performed mostly on arbuscular plants confirmed that individual plants performed best at the lowest plant densities and competed severely for nutrients when growing at higher densities (Koide 1991; Facelli et al. 1999). For example, Facelli et al. (1999) showed that *Trifolium subterraneum* plants had lower biomass when growing at higher densities; however, there was no significant effect of fungal inoculum on plant biomass at higher densities. Hartnett et al. (1993) showed that negative plant responses to arbuscular fungal colonization were noticeable for single plants or at low density, but were reduced in some cases at high plant density. This may be explained by a greater overlap of roots and successful competition and, hence, better acquisition of soil resources by larger nonmycorrhizal plants. Cheng and Bledsoe (2004) demonstrated that the grass *Avena barbata* growing at high density limited nitrogen availability for both co-occurring *Quercus douglasii* and soil microorganisms, suggesting a strong effect of interspecific competition and an advantage for the grass in nutrient acquisition. Archer and Detling (1984) observed that plants growing in competition responded with lower biomass and flowering, regardless of whether or not they were subjected to defoliation. This implies that the effects of competition might be more harmful for plant growth than aboveground injury. It is important to note that damaged plants could still compete efficiently with their neighbors for soil resources. Defoliation may hinder root development and in turn limit water and nutrient uptake (Evans 1972). However, defoliation does not always hinder nutrient acquisition, as shown by Chapin and Slack (1979).

We used tree species in our study that differ in their light requirements: shade-tolerant *Fagus sylvatica* (Minotta and Pinzauti 1996) and light-demanding *P. sylvestris* (Pearcy et al. 2004). The species respond differently to irradiance, with light-demanding *P. sylvestris* showing more pronounced changes in leaf morphology and thus better light exploitation than *F. sylvatica* (De Chantal et al. 2003). Shade-tolerant trees are better adapted to shade because they can survive light limitations in their juvenile period (Kitajima 1994).

This research was designed to study the responses of light-demanding pioneer *P. sylvestris* and shade-tolerant *F. sylvatica* seedlings to interactive effects of partial foliage and/or root loss, light limitations, and interspecies competition on mycorrhizal root tip colonization, growth, and seedling mineral status. We tested the following hypotheses: (1) carbohydrate shortage under low-light conditions or damage treatments that simulate animal grazing will result in a decrease of growth, ECM root tip colonization, and leaf nitrogen content; (2) competition with grasses will decrease ECM root tip colonization, seedling growth, and leaf nitrogen content; (3) high-light conditions will reduce the destructive effects of both damage treatments (hypothesis 1) and competition (hypothesis 2); and (4) the above responses will be tree species dependent and *Pinus* seedlings will suffer more than *Fagus* seedlings under low light.

## Material and methods

### Plant material

Seeds of *F. sylvatica* and *P. sylvestris* were purchased from the Jarocin Forest District Seed Bank (Poland). The pine seeds were directly planted without any preliminary preparation, while beech seeds (in nuts) underwent cold stratification (3 °C) for 10 weeks (i.e., until the appearance of the first germinated seeds). Seeds were screened for their mass and those of medium weights were planted into pots containing peat and perlite (1:1, *v*/*v*). Before planting into pots, seed germination was tested. Germinating seeds were treated with the fungicide Previcur 607 SL (Bayer) at 15 % concentration. Young seedlings (just after cotyledon development) were carefully removed from the pots and subjected to leaf and root damage treatments simulating animal grazing (Table 1).

**Table 1:** A set of juvenile damage treatments for *Fagus sylvatica* and *Pinus sylvestris* seedlings used in the experiment

Treatment no.	Description
*Fagus sylvatica*	
1	1 cotyledon removed
2	1 cotyledon and 20 % of the root by length removed
3	20 % of the root by length removed
4	Control (intact)
*Pinus sylvestris*	
1	1 cotyledon removed
2	1 cotyledon and 20 % of the root by length removed
3	3 cotyledons removed
4	3 cotyledons and 20 % of the root by length removed
5	5 cotyledons removed
6	5 cotyledons and 20 % of the root by length removed
7	20 % of the root by length removed
8	Control (intact)

Seedlings of each treatment were then planted into larger pots (5 L). Soils were collected from monoculture *P. sylvestris* and *F. sylvatica* stands near Kórnik from the upper ca 20 cm after litter removal. Before seed planting, the soils were mixed together with peat (1:1:1, *v*/*v*/*v*) and enriched by inorganic fertilizer (3 kg/1000 L) (Osmocote Exact, Scotts).

#### Experimental design

Seedlings were grown in shade tents made of polypropylene netting (Agrotex, Poland). On a cloudy day in April 2005, the photosynthetic photon flux density (PPFD) was simultaneously measured in the open and in a tent using linear photon detectors (Apologe Inc., USA). Then, for both shade treatments, the mean percent of full sunlight (relative photosynthetic photon flux density—rPPFD) was calculated. Two shade treatments were established: low light (5 % of full irradiance) and high light (50 %). These light regimes, low and high, used in the experiment, simulated the natural conditions of *Fagus* and *Pinus* seedlings grown under the dense forest canopy and openings, respectively.

In total, there were 60 pots randomly arranged in four blocks for each damage treatment under both light regimes. Three seedlings were grown in each pot. In the competition treatment, 20 transplanted grass individuals (*Bromus sterilis* L.) collected in early May in 2005 in the Kórnik Arboretum were used in each pot containing three seedlings of either *F. sylvatica* or *P. sylvestris* seedlings. For the competition experiment, we used 24 pots for each tree species, damage treatment, and light regime. After seeds of the grass developed (midsummer) and started germinating in the pots, the seedlings were carefully removed to maintain the fixed number of grass individuals.

#### Ectomycorrhizal, morphological, and chemical characteristics of seedlings

*Fagus* and *Pinus* seedlings were harvested monthly from July to November 2005. Eight seedlings of each damage treatment from both light conditions were used to determine whole plant as well as root, shoot, and leaf biomass; morphology of leaves and roots; and nitrogen, carbon, and carbohydrate contents of plants. Additionally, three to four seedlings from each treatment were used to assess ectomycorrhizal root tip abundance based on microscopic observations. Three to four seedlings growing in control or in competition with grasses per damage and light treatment were also collected in early September 2005 to quantify ectomycorrhizal colonization.

The extent of the ECM colonization was determined using a stereomicroscope. Ectomycorrhizal and nonmycorrhizal root tips were assessed, and the extent of the ECM colonization was expressed as a percentage of ECM root tips among all root tips. For seedlings containing only a few ECM root tips, relative abundance was estimated as 1 %. Identifications of ectomycorrhizal morphotypes were based on a standard rDNA-internal transcribed spacer (ITS) barcoding procedure (Trocha et al. 2012). Shortly after microscopic examination, total genomic DNA was extracted using a Plant & Fungi DNA Purification Kit (EURx, Poland). Internal transcribed spacer regions (ITS1-5.8S-ITS2) were amplified via the polymerase chain reaction (PCR) using the primers fungal specific ITS1-F and universal ITS4. The PCR reactions were performed in a 10-μL volume mixture consisting of 1× PCR buffer (Novazym, Poland), 1.5 mM MgCl_2_ (Novazym, Poland), 0.2 mM of each dNTP (Novazym, Poland), 0.5 μM of each primer, 0.02 mg mL^−1^ BSA (Promega, Madison, WI, USA), 0.25 U of *Taq* Polymerase (Novazym, Poland), and 5 μL of DNA aliquot. Reactions were performed in a thermocycler using the following temperature profiles: 1 min 93 °C (initial denaturation), 1 min 95 °C (denaturation), 1 min 60 °C (annealing), 2 min 72 °C (elongation), 10 min 72 °C (final elongation), and 7 °C (pause). Steps 2 to 4 were repeated 35 times. PCR products were directly sequenced using primers ITS1 and/or ITS4. DNA sequencing was undertaken using the ABI Prism Big Dye Terminator Sequencing Kit version 3.1 (Applied Biosystems, Foster City, CA, USA). Cycle sequencing was performed using a 2720 Thermal Cycler followed by capillary electrophoresis in an ABI Prism 3130XL Genetic Analyzer (Applied Biosystems, Foster City, CA, USA). The ITS sequences of all morphotypes were compared to sequences in GenBank using the *blastn* algorithm and then aligned to their best GenBank matches using CodonCode Aligner (http://www.codoncode.com/aligner) at the 97 % identity threshold. The obtained sequences of fungal taxa were deposited in GenBank (accession numbers KP857984–KP857995).

### Statistics

The data were analyzed using three-way analysis of variance (ANOVA) with light, competition, and damage treatments as the main factors and block as a random factor. Prior to ANOVA, homogeneity of variances was tested with Levene’s test and normality of distributions with the Shapiro-Wilk test. The main effects ANOVA was applied to test for differences in the mean values of the measured parameters among blocks, damage treatments, competition, and light regimes at *p* < 0.05. Then, the full factorial ANOVA was applied (Tables 2, 3, and 4). When the block effect was not significant in the main effects ANOVA, it was excluded from the ANOVA model. The following were the levels in each main factor: light—two levels (low light, high light), competition—two levels (no competition, competition), and damage treatment called “further treatment”—four levels in *Fagus* and eight levels in *Pinus* (see Table 1). ANOVA was conducted separately for *Fagus* and *Pinus*.

**Table 2:** The results of ANOVAs for ectomycorrhizal tip abundance (ECM tip abundance (%)), seedling biomass (g DW), and leaf N content (mg N g^−1^ DW) of *Fagus sylvatica* (Fs) and *Pinus sylvestris* (Ps) injured mechanically (treatment), growing in low light or high light (light) and in competition with grasses or not (competition)

	Host	Trait	Treatment		Light		Treatment × light		Competition		Treatment × competition		Light × competition		Treatment × light × competition	
			*df*	*F*	*df*	*F*	*df*	*F*	*df*	*F*	*df*	*F*	*df*	*F*	*df*	*F*
1	Fs	ECM tip abundance (%)	3	2.91*	1	88.85***	3	2.24 n.s.								
2	Ps		7	1.99 n.s.	1	1010***	7	1.99 n.s.								
3	Ps								1	24.4***						
4	Fs								1	0.0024 n.s.						
5	Fs	Seedling biomass (g)	3	14.2***	1	618***	3	2.05 n.s.	1	123***	3	0.88 n.s.	1	57.1***	3	1.00 n.s.
6	Ps		7	8.94***	1	1415***	3	1.34 n.s.								
7	Ps					n.a.			1	327***						
8	Fs	Leaf N (mg g^−1^)	3	2.17 n.s.	1	25.6**	3	1.15 n.s.	1	30.2***	3	0.81 n.s.	1	0.17 n.s.	3	0.41 n.s.
9	Ps		7	3.05*	n.a.		n.a.		1	48.2***	7	1.88 n.s.	n.a.		n.a.	

Degree of freedom (*df*) and *F* values are given

*n* *.a.* not available, *n.s.* not significant

***p < 0.0001; **0.001 > *p* ≥ 0.0001; *0.05 > *p* ≥ 0.001

**Table 3:** Means (±SE) of ectomycorrhizal (ECM) root tip abundance, seedling biomass, and leaf N of *Fagus sylvatica*, growing in low light or high light (light) and in competition with grasses or not (competition); the mean values followed by the same letter are not significantly different in ANOVA (light, competition) or Tukey’s a posteriori test (treatment) (*p* < 0.05)

Trait	Harvest	Number	Treatment				Light		Competition	
			1	2	3	4	Low	High	No	Yes
ECM tip abundance (%)	November	64	10 (±3)b	15 (±6)ab	18 (±7)ab	29 (±10)a	0.3 (±0.1)b	36 (±6)a		
	September	88							18 (±6.5)a	13 (±6.6)a
Seedling biomass (g)	November	196	1.1 (±0.2)ab	1.0 (±0.2)b	1.4 (±0.2)ab	1.6 (±0.3)a	0.4 (±0.02)b	2.1 (±0.2)a	2.2 (±0.3)a	0.8 (±0.1)b
Leaf N (mg g^−1^)	November	32	22 (±1.4)a	25 (±0.9)a	24 (±1.4)a	25 (±1.5)a	26 (±0.8)a	22 (±0.8)b	22 (±0.8)b	26 (±0.7)a

**Table 4:** Means (±SE) of ectomycorrhizal (ECM) root tip abundance, seedling biomass, and leaf N of *Pinus sylvestris* injured mechanically (treatment), growing in low light or high light (light) and in competition with grasses or not (competition); the mean values followed by the same letter are not significantly different in ANOVA (light, competition) or Tukey’s a posteriori test (treatment) (*p* < 0.05)

Trait	Harvest	Number	Treatment								Light		Competition	
			1	2	3	4	5	6	7	8	Low	High	No	Yes
ECM tip abundance (%)	September	85											0.5 (±0.1)b	2.8 (±0.6)a
	November	128	46 (±12)a	46 (±12)a	36 (±11)a	35 (±11)a	36 (±11)a	46 (±12)a	46 (±12)a	47 (±12)a	1 b	83 (±3.0)a		
Seedling biomass (g)	November	155											0.9 (±0.1)a	0.12 (±0.01)b
	November	110	0.6 (±0.2)ab	0.6 (±0.2)ab	0.4 (±0.1)b	0.5 (±0.1)b	0.2 (±0.04)c	0.5 (±0.1)b	0.7 (±0.2)a	0.5 (±0.1)a	0.04 (±0.002)b	0.9 (±0.06)a		
Leaf N (mg g^−1^)	November	32	21 (±1.3)a	22 (±0.6)a	22 (±0.7)a	21 (±1.4)a	23 (±1.8)a	23 (±1.2)a	22 (±0.7)a	22 (±0.5)a	n.a.	n.a.	24 (±0.4)a	21 (±0.3)b

The mean values within the treatments were compared with Tukey’s a posteriori test at the significance level *α* = 0.05. Prior to ANOVA, the data expressed in percent were transformed using the Bliss function: arcsine((ECM root tip %/100)^0.5^) * 180/3.14 and seedling biomass and leaf N data were log transformed prior to ANOVA. All statistical analyses were conducted with JMP v. 8 (SAS Package, USA).

## Results

### ECM root tip abundance and seasonal changes in EMF composition

Significant differences of ECM root tip abundance (%) were found among the damage treatments and the control in November (Table 2), when we observed the lowest ECM root tip abundance in the seedlings of treatment 1 (one cotyledon removed, mean 10 %) and the highest in control seedlings (mean of 29 %) (Table 3). Competition with grasses showed no effect on ECM root tip abundance (Table 3).

**Fig. 1:**
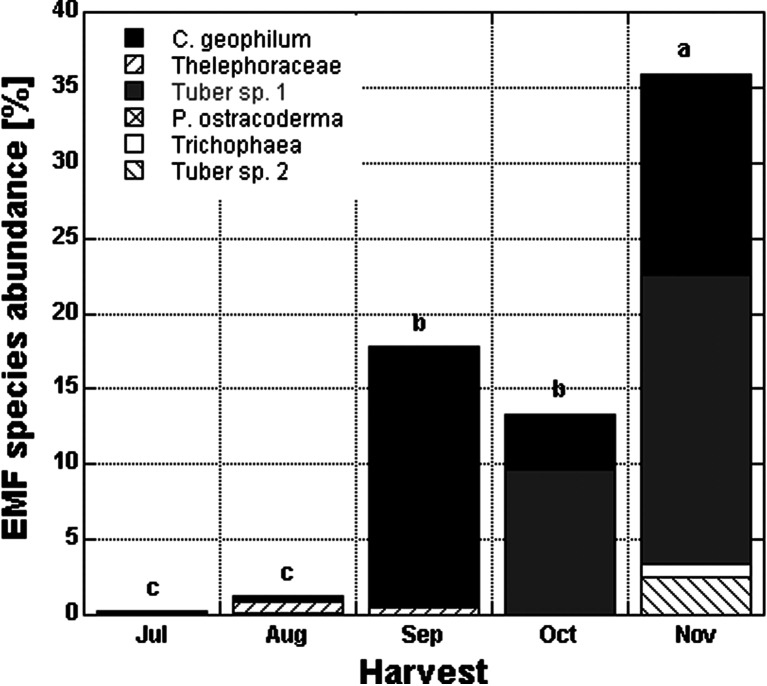
Mean ECM root tip abundance (%) and ectomycorrhizal fungal species composition in *Fagus sylvatica* seedlings over time for ca 30-day intervals. All damage treatments are included for the high light and no competition treatments (*n* = 160, *n* = 32 for each harvest). *Levels not connected by the same letter* are significantly different

During the season, the mean ECM root tip abundance of *Fagus* seedlings growing under high light increased from a mean of 0.65 % in July to 36 % in November (Fig. 1). Seedlings growing under high light collected in the final harvest (November) had ca 120 times higher ECM root tip abundance than those growing under low light (Table 3). Ectomycorrhizal fungus species composition in *Fagus* seedlings collected over a season revealed six EMF taxa in total (Fig. 1). The most frequent EMF species was *Cenococcum geophilum*, found in the seedlings collected in each harvest, followed by *Tuber* sp. 1 and *Thelephoraceae* (Fig. 1).

*Pinus* seedlings collected in November from both light regimes also showed a strong decrease of ECM root tip abundance under low light (ca 1 %) compared to those growing under high light (83 %) (Table 4). *Pinus* seedlings collected in September from the competition treatment showed a strong effect of competition on ectomycorrhizal colonization (Table 2) with a higher ECM root tip abundance in seedlings growing in competition with grasses (ca 3 %) than those growing alone (0.5 %) (Table 4).

**Fig. 2:**
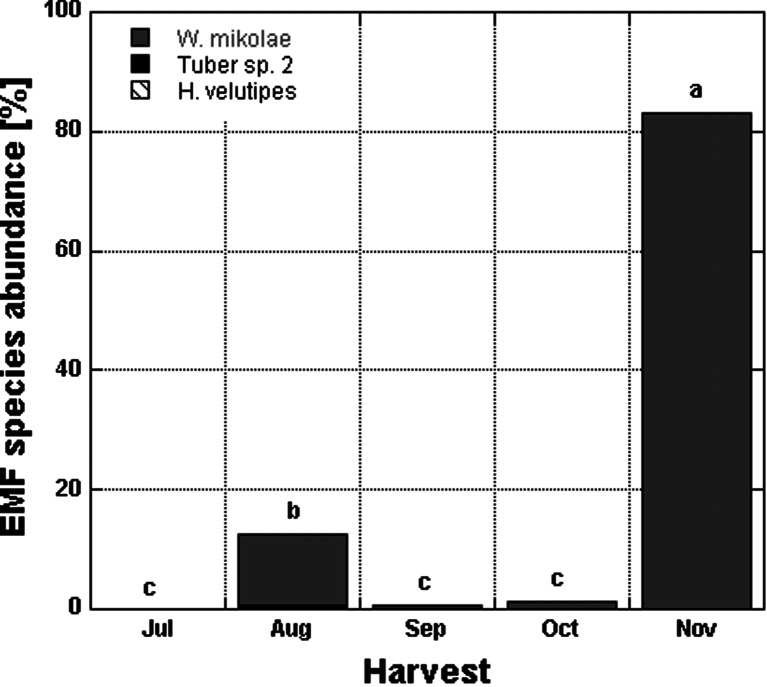
Mean ECM root tip abundance (%) and ectomycorrhizal fungal species composition in *Pinus sylvestris* seedlings over time for ca 30-day intervals. All damage treatments are included for the high light and no competition treatments (*n* = 319, *n* = 63–64 for each harvest). *Levels not connected by the same letter* are significantly different

Ectomycorrhizal colonization in *Pinus* followed a similar pattern as *Fagus* and showed an increase over a season starting from the lowest of 0.05 % in July to the highest of 83 % in November (Fig. 2). However, contrary to *Fagus* seedlings, ectomycorrhizal colonization of *Pinus* seedlings collected in November did not differ significantly among the damage treatments (Tables 2 and 4).

Ectomycorrhizal species identification revealed three EMF taxa colonizing *Pinus* roots collected over a season. Root tips of *Pinus* seedlings were dominated by the ECM fungus *Wilcoxina mikolae*, while *Hebeloma velutipes* and *Tuber* sp. 1 occurred occasionally at low percentages (Fig. 2).

### Seedling biomass and leaf nitrogen contents

Juvenile damage treatment had a significant effect on seedling biomass collected in November (Table 2). The lowest biomass was for seedlings that had the most foliage removed: five out of eight cotyledons removed (*Pinus*, treatment no. 5) (Table 4), but *Fagus* seedlings did not reveal such a pattern (Table 3).

Light affected seedling biomass significantly (Table 2). We found that *Fagus* seedlings growing under low light had ca 5 times lower biomass (Table 3) than those growing under high light, while *Pinus* seedlings had an even greater response—biomass of seedlings growing under low light was 22 times lower compared to seedlings growing under high light (Table 4).

**Fig. 3:**
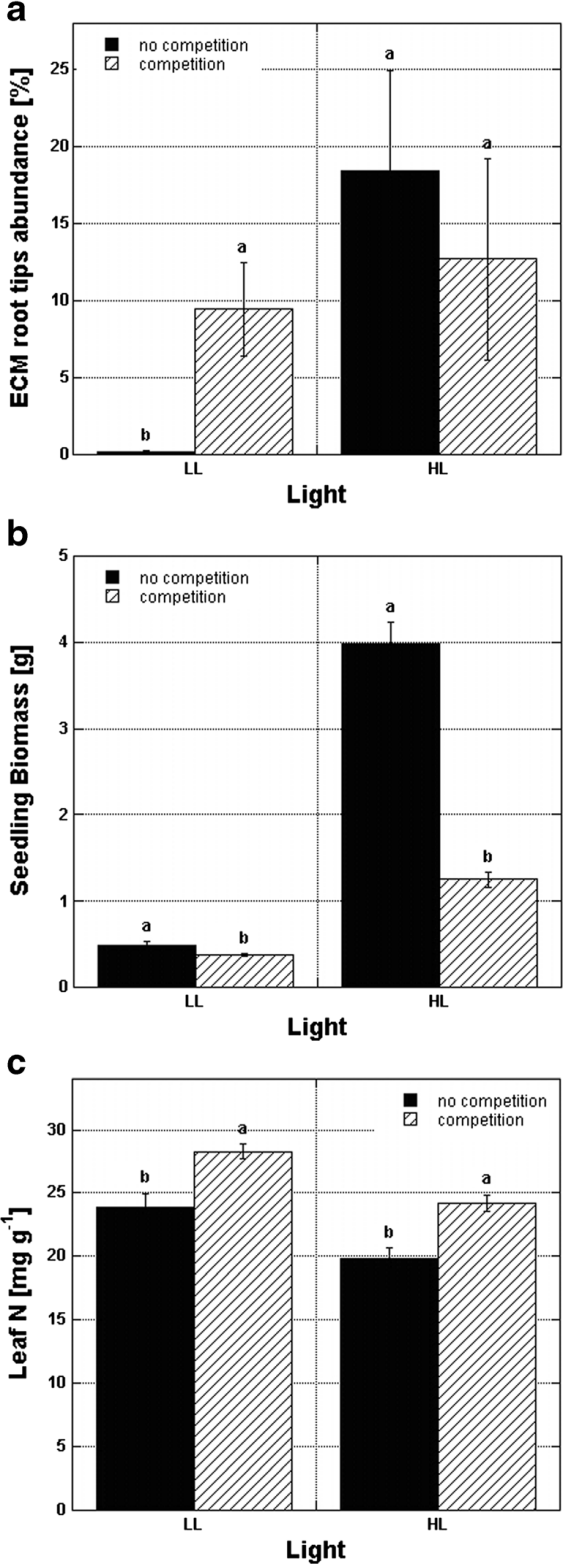
Effect of competition within light regimes on ECM root tip abundance (**a**), seedling biomass (**b**), and leaf N (**c**) of *Fagus sylvatica*; *LL* low light, *HL* high light; *levels not connected by the same letter* are significantly different between competition treatments within each light regime; *n* = 88 (**a**), *n* = 196 (**b**), and *n* = 32 (**c**)

*Fagus* and *Pinus* mean seedling biomass was 2.7 and 7.5 times lower, respectively, when seedlings grew with grasses compared to seedlings growing without grasses (Tables 3 and 4). Depending on the light regime, the effect of competition was partially reduced as shown for *Fagus* ECM root tip colonization (Fig. 3a) and seedling biomass (Fig. 3b); however, high light did not improve leaf N content (Fig. 3c).

Leaf nitrogen content showed no differences among the damage treatments in *Fagus*, but it did in *Pinus* (Table 2). Moreover, significant effects of light conditions and competition were found (Table 2). *Fagus* seedlings growing in low light had ca 1.2 times more leaf N than those growing in high light (Table 3). The analysis was not available for *Pinus* seedlings as there was no sufficient number of replicates from low light (data not shown). Competition with grasses also influenced leaf N in both tree species (Table 2). For *Fagus*, seedlings growing in competition revealed higher leaf N than those growing alone (Fig. 3c; Table 3). However, in *Pinus*, we observed the opposite trend with higher leaf N for seedlings growing alone (Table 4).

## Discussion

Trees growing in natural conditions are constantly subjected to a vast number and different types of threats; some of those are mechanical (animal grazing), while others constitute interactions with other plants including competition for soil resources and light. Knowledge of the extent to which seedlings may recover after being affected by biotic and/or abiotic threats at a juvenile stage of growth is limited (Longer and Oosterhuis 1999). The fate of a plant in natural conditions where many uncontrolled stress factors may occur is difficult to study.

Consistent with several studies on herbaceous (Gehring 2003 and literature cited therein) and woody plants (Ekwebelam and Reid 1983), mycorrhiza formation increased in high-light conditions in both *Fagus* and *Pinus* seedlings (Tables 3 and 4; Fig. 3a). Both tree species had higher biomass when growing under high light, but leaf N was lower in *Fagus* (*Pinus* not available) (Table 3). Markkola et al. (2004) and Saravesi et al. (2008) demonstrated that carbohydrate shortage due to defoliation resulted in a decrease of high-biomass ectomycorrhizal fungus species, suggesting that they require relatively large amounts of carbon. We did not find changes in the composition of EMF communities associated with *Fagus* or *Pinus* due to light or damage treatments, which was opposite to the results of Markkola et al. (2004) and Saravesi et al. (2008). *C. geophilum* and *Tuber* spp. were the dominant EMF of *Fagus* (Fig. 1), both displaying thick mantles. According to the descriptions of Saravesi et al. (2008), these morphotypes have rather high biomass and, thus, may require more carbon as has been shown for respiration of thick-mantle morphotypes (Trocha et al. 2010). For *Pinus*, the thin-mantled *W. mikolae* was the dominant EMF (Fig. 2), and light limitation did not change the EMF composition (data not shown).

A high mycorrhizal root tip abundance may promote several plant responses, including higher stomatal conductance (Augé et al. 2004), improved plant-water relations (Muhsin and Zwiazek 2002; Shi et al. 2002; Kennedy and Peay 2007), more effective nutrient acquisition (Wallander et al. 1994; Wallander and Hagerberg 2004), greater resistance to fungal pathogens (Branzanti et al. 1999), and higher photosynthetic activity (Dosskey et al. 1990). Growing in a well-mixed fungal community (pot substrate containing a forest soil mix), seedlings were subjected to both beneficial and less beneficial EMF species in terms of carbon costs to the hosts (Bever et al. 2009). EMF communities did not, however, respond to light or damage treatments. Plant biomass and ECM root tip abundance of either *Pinus* or *Fagus* were affected negatively by low light, indicating light as a factor of high importance for plant and ECM fungi niche partitioning (Brearley et al. 2007). The dominance of Ascomycota colonizing the roots of *Fagus* and *Pinus* seedlings in our experiment is difficult to explain: it could result from disturbed (mixed) soil or carbon demands of the fungi/amounts of carbon allocated to roots in very young seedlings. It has been shown that the species of Ascomycota (mostly *Wilcoxina* sp.) dominated in the seedlings of conifer tree species grown under nursery conditions (Rudawska et al. 2006; Trocha et al. 2006; Leski et al. 2008). However, Basidiomycota dominated in other tree seedlings, including conifers, growing under similar nursery conditions (Leski et al. 2010; Menkis and Vasaitis 2011). We think that this issue needs more attention in future research.

Defoliation of a very young seedling may trigger different compensatory mechanisms, including an increase in photosynthetic activity in the remaining leaves (Mabry and Wayne 1997), foliage increase (Khan et al. 2002), and increase in mineral nutrient uptake (Ruess 1984), which, in turn, may cause better plant growth (McNaughton 1983). However, numerous papers report a negative effect of defoliation on seedling survival (e.g., von Schütt 1973; Myers 1981; Wilkinson and Nielsen 1995; Muro et al. 1998; Li and Ma 2003). Our results support the latter finding: the biomass of 6-month-old *Pinus* and *Fagus* seedlings was influenced negatively by juvenile damage (Tables 3 and 4; Fig. 3b). The most significant response that resulted in the lowest mean ECM root tip abundance and biomass was caused by the most intensive damage: removing five (out of eight) cotyledons in *Pinus* (Table 4), suggesting that photosynthesis shortage is a determining factor in plant functioning. However, when ECM root tip abundances among damage treatments were analyzed separately under the light regimes (not shown), organ loss did not affect ectomycorrhizal colonization, suggesting that light is a predominant factor influencing plant physiology.

Interspecific competition is one of the most important forces shaping the diversity of plant communities and total aboveground plant biomass (Del-Val and Crawley 2005). The effect of competition on ECM root tip abundance was different between the two tree species studied (Tables 3 and 4). Competition with arbuscular-mycorrhizal shrubs decreased both biomass and ectomycorrhizal colonization in *Pinus edulis*, while in our research, we noticed that competition promoted ECM root tip colonization in *Pinus* seedlings, but not in *Fagus*, and simultaneously decreased their biomass (Tables 3 and 4). That is striking, as many researchers have observed that competition negatively impacted ECM root tip colonization in many ecosystems (McHugh and Gehring 2006 and literature cited). Light availability promoted ectomycorrhizal colonization (Fig. 3a) and seedling biomass in the “no competition” treatment (Fig. 3b), again suggesting light’s predominant role in plant functioning. However, Cheng and Bledsoe (2004) found a negative effect of grass both underground and aboveground that reduced *Quercus* seedling growth and soil nitrogen availability. The impact of grasses could be both direct (competing for soil resources with other plants) and indirect—grasses may change the microclimate around seedlings in a way that hinders growth (Ball et al. 2002).

We clearly showed that light availability is the main factor affecting seedling growth and ectomycorrhizal root tip colonization in our study. High light (50 % of full irradiance) compensated to some extent for the negative effects of organ loss and competition with grasses on ectomycorrhizal colonization and growth of *P. sylvestris* and *F. sylvatica* seedlings.
